# *Panicovirus *accumulation is governed by two membrane-associated proteins with a newly identified conserved motif that contributes to pathogenicity

**DOI:** 10.1186/1743-422X-3-12

**Published:** 2006-03-08

**Authors:** Jeffrey S Batten, Massimo Turina, Karen-Beth G Scholthof

**Affiliations:** 1 Department of Plant Pathology and Microbiology, Texas A&M University, College Station, TX, USA; 2 G.C. Hawley Middle School, Creedmoor, NC, USA; 3 Istituto di Virologia Vegetale, Torino, Italy

## Abstract

*Panicum mosaic virus *(PMV) has a positive-sense, single-stranded RNA genome that serves as the mRNA for two 5'-proximal genes, p48 and p112. The p112 open reading frame (ORF) has a GDD-motif, a feature of virus RNA-dependent RNA polymerases. Replication assays in protoplasts showed that p48 and p112 are sufficient for replication of PMV and its satellite virus (SPMV). Differential centrifugation of extracts from PMV-infected plants showed that the p48 and p112 proteins are membrane-associated. The same fractions exhibited RNA polymerase activity *in vitro *on viral RNA templates, suggesting that p48 and p112 represent the viral replication proteins. Moreover, we identified a domain spanning amino acids 306 to 405 on the p48 and p112 PMV ORFs that is common to the *Tombusviridae*. Alanine scanning mutagenesis of the conserved domain (CD) revealed that several substitutions were lethal or severely debilitated PMV accumulation. Other substitutions did not affect RNA accumulation, yet they caused variable phenotypes suggestive of plant-dependent effects on systemic invasion and symptom induction. The mutants that were most debilitating to PMV replication were hydrophobic amino acids that we hypothesize are important for membrane localization and functional replicase activity.

## Introduction

**Figure 1:**
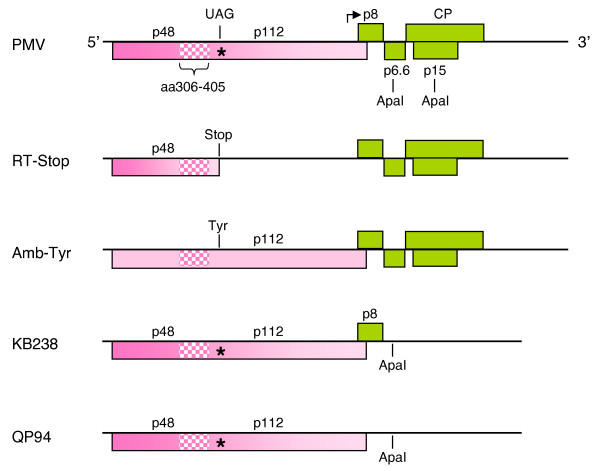
**PMV genome and cDNA mutants for replication assays**. The PMV genome map shows six open reading frames (ORFs), as filled rectangles. The solid line represents the 4,326 nucleotide single-stranded plus-sense PMV genomic RNA (gRNA). Four proteins (p8, p6.6, p15, and CP) are encoded from the subgenomic RNA (sgRNA), which initiates at nucleotide 2851 (bent arrow). Both p48 and p112 are expressed from the gRNA from an AUG start codon at nt 29–31. The UAG (amber) read-through codon is indicated with an asterisk (*). The speckled region indicates the conserved domain (CD), from amino acids 306–405, encoded on both p48 and p112. Two replicase mutants, pRT-Stop and pAmb-Tyr, express p48 or p112, respectively. The *Apa*I sites were used to delete nucleotides 3129 to 3400 on the PMV cDNA. This deletion abolished the expression of p6.6, p15, and CP on the sgRNA to create pKB238. Another construct, pMAX6 [18] with a point mutation to abolish translation of the p8 ORF, was digested with *Apa*I and religated. This created pQP94, a construct that no longer expressed any of the sgRNA encoded genes.

*Panicum mosaic virus *(PMV), a 4.3 kb positive-sense ssRNA virus, is the type member of the *Panicovirus *genus in the *Tombusviridae *[1,2]. Like other members of this family [3,4], PMV encodes two proteins expressed from the 5'-proximal half of the ssRNA genome (Fig. 1). For most members of the *Tombusviridae *the first open reading frame (ORF) encodes a protein of approximately 25–30 kDa. In contrast, the molecular weight of the PMV 5'-proximal encoded protein is considerably higher (48 kDa). Similarly, a second protein that is expressed as a translational read-through product usually generates an 80 to 100 kDa protein; instead, PMV encodes a protein of 112 kDa. In all cases, the downstream portion of the larger translational product contains the GDD-motif, a characteristic feature of RNA-dependent RNA polymerases [5]. A unique combination of properties of PMV is that it infects monocots and it supports the replication and movement of three different types of subviral agents. PMV serves as the helper for a satellite virus (SPMV), satellite RNAs and an SPMV-derived defective interfering RNA (DI) [1,6-9].

For some members in the *Tombusviridae *it is known that on the gRNA, the 5'-proximal encoded protein and its translational read-through product are membrane-associated replicase proteins [10-16]. However, this has not yet been demonstrated for p48 and p112 encoded by PMV. In an earlier report, we identified a series of amino acids conserved in the N-proximal replicase-associated proteins in members of the *Tombusviridae *[17]. This conserved domain (CD) is located between amino acids 313 to 405 on PMV p48 and p112. These considerations provided the context for the following five interrelated objectives of the present study. The first aim was to determine if p48 and p112 were membrane-associated and if these fractions contained RNA polymerase activity. The second goal was to examine if p48 and p112 are the only viral proteins required for replication of PMV and SPMV. Thirdly, we investigated if p48 is required for replication. The fourth objective was to examine the contribution of the CD to replication of PMV and SPMV. The fifth aim was to evaluate if amino acids in the CD had additional pathogenicity properties.

The results show that both p48 and p112 co-fractionate with membranes and these fractions have *in vitro *RNA polymerase activity. Replication assays with site-directed mutants showed that both proteins are required and sufficient for PMV and SPMV replication. Some amino acid substitutions on the CD abolished replication of PMV and SPMV, whereas others caused a reduction and delay in symptom development.

## Results

### PMV p48 and p112 proteins are required for PMV replication

**Figure 2:**
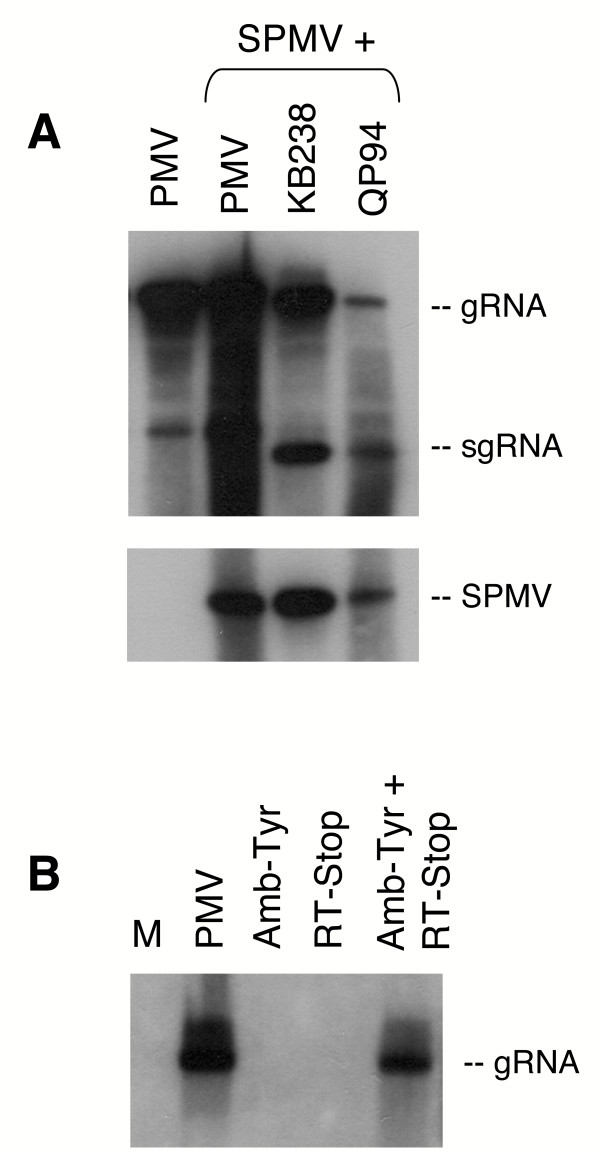
**Transfected foxtail millet protoplasts were harvested 2 days post-inoculation**. **(A) **PMV transcripts inoculated alone or with SPMV. Transcripts KB238 and QP94 were co-inoculated with SPMV transcripts. The RNA was extracted and separated on TBE-agarose gels, blotted, and probed for PMV or SPMV accumulation with a ^32^P-labelled cDNA to detect PMV genomic (g) and subgenomic (sg) RNAs or SPMV RNA, respectively. **(B) **RNA blot of total RNA isolated from protoplasts inoculated with RT-Stop, Amb-Tyr, or both (RT-Stop + Amb-Tyr). Detection of PMV was as described for Panel A. PMV was used as a positive control and M represents mock-inoculated protoplasts.

Protoplast assays with transcripts from the PMV mutant pQP94, that expresses only p48 and p112 (Fig. 1), showed readily detectable levels of gRNA replication, production of sgRNA, and replication of SPMV *in trans *(Fig. 2A). In contrast, PMV-derived transcripts of mutants separately expressing p48 (pRT-Stop) or p112 (pAmb-Tyr), did not replicate (Fig. 2B). Therefore, all four genes expressed from the sgRNA are dispensable for virus replication. However, comparison of RNA accumulation between KB238 and QP94 indicates that the expression of p8 may enhance the levels of RNA accumulation. As for PMV, the replication of SPMV also required the expression of both p48 and p112 replication (Fig. 2A and data not shown).

PMV transcripts expressing either p48 (from pRT-Stop) or p112 (from pAmb-Tyr) did not accumulate detectable levels of gRNA in protoplasts, yet co-transfection with pRT-Stop + pAmb-Tyr transcripts restored PMV replication (Fig. 2B). This is most likely due to *trans*-complementation rather than *in vivo *recombination to the wild-type genotype because the identical mixed-inoculations on plants did not establish infections (data not shown). Collectively, these data show that p48 and p112 are both necessary and sufficient for replication of PMV and SPMV RNAs.

### Membrane-associated RdRp activity

**Figure 3:**
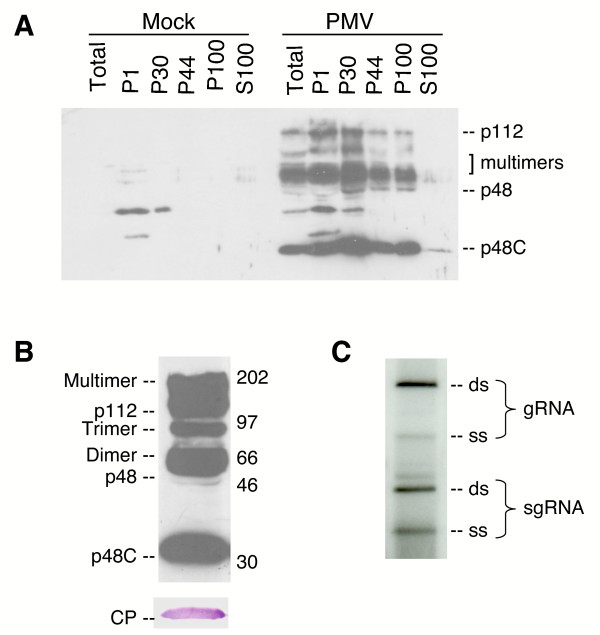
**Subcellular fractionation and analyses of the PMV replicase proteins isolated from millet plants and assay for RNA products generated by the PMV RNA-dependent-RNA polymerase (RdRp)**. **(A) **Mock-inoculated and PMV-infected leaves were harvested and subjected to differential centrifugation to isolate the cell wall, nuclei and chloroplasts (P1), membranes (P30, P44, P100), and soluble proteins (S100). Proteins were separated by SDS-PAGE, transferred to nitrocellulose membrane, and probed with rabbit polyclonal antiserum against the C-terminal half of p48 (p48C). **(B) **The P44 (44,000 × *g*) fraction isolated from PMV-infected millet plants probed with p48C-derived antiserum (upper panel) or CP-specific antiserum (lower panel). The molecular weight markers are indicated in kDa, and the predicted forms of the PMV RdRp encoded proteins (p48 and p112) are shown, including the 48C (~29 kDa) derivative and its putative dimer, trimer, and multimeric forms. **(C) **The P44 fraction from PMV-infected plants was assayed for *in vitro *RdRp activity measured by incorporation of [^32^P]-UTP into the associated PMV RNAs. The products were analyzed on TBE-agarose gels followed by transfer to nylon membranes and exposure to X-ray film. The single stranded- (ss) and double-stranded (ds) genomic (gRNA) and subgenomic (sg) RNAs are indicated.

Differential centrifugation of extracts from healthy and PMV-infected millet plants followed by immunoblot assays demonstrated that p48 and p112 were predominant in membrane-enriched fractions of infected plants (Fig. 3A). The P44 fraction (44,000 × *g *pellet) was selected for further assay as it exhibited the least amount of host protein (data not shown). Immunoblot assays using antiserum derived from the C-terminal half of p48, detected the predicted p48 and p112 proteins. The polyclonal antibody also detected a 30 kDa protein (p48C; Fig. 3A), and its ~60 kDa dimer. The p48C protein is predicted to represent the C-terminal portion of the p48 protein (see Discussion). The two ~100 kDa proteins, may be p112 and multimers of p48C (~120 kDa). PMV CP was also consistently associated with membrane-enriched fractions (Fig. 3B), suggesting it may have a role as a co-factor for replication or cellular localization [18]. We have also detected a complex of PMV replicase proteins and the CP by fractionation on a 75 cm Sephacryl column [19]. These data show PMV is similar to other plant viral RNA-dependent RNA polymerase proteins in that the replicase is associated with cellular membranes.

The 44,000 × *g *(P44) enriched membrane fraction was mixed with ribonucleotides and [^32^P]-UTP in reaction buffer to assay RdRp activity. Newly synthesized RNA products were detected in reactions containing the P44 fraction from PMV-infected plants (Fig. 3C) but not from mock inoculated plants (data not shown). The majority of the products on the RNA blot were double-stranded (ds) forms of gRNA and sgRNA while ssRNAs were detected as less intense bands (Fig. 3C). The two major products were not susceptible to S1 nuclease treatment confirming their double-stranded nature and showing that the RNA was not merely end-labeled by terminal transferase activity (data not shown) [19]. These experiments agreed with the prediction that PMV replication occurs in association with membranes and that the p48 and p112 proteins (and perhaps CP) are key virus functional elements in this process.

### A conserved domain (CD) in the replicase proteins of *Tombusviridae*

BLAST analysis of the PMV p48 ORF (that also represents the 5'-proximal half of p112) showed that it is related to other 5'-proximal ORFs from members of the family *Tombusviridae *[2]. However, reiterative pair-wise comparisons using BLAST-PSI of the full-length p48 coding sequence to similar replicase-associated proteins yielded a relatively low percent identity (3 to 19%). The highest number of comparative identical amino acids (19%) was to the 50 kDa replicase protein of *Maize chlorotic mottle virus *(MCMV; genus *Machlomovirus*)[20].

**Figure 4:**
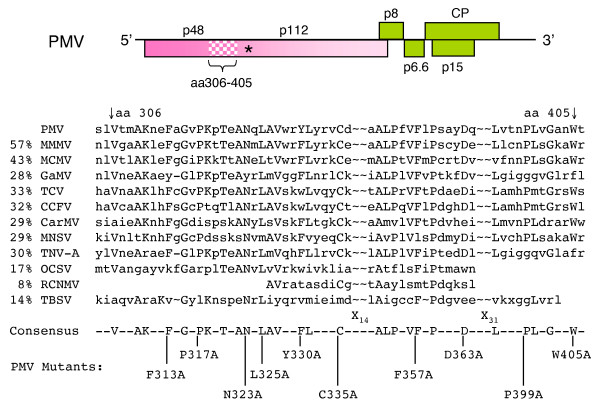
**Replicase motif conserved in the *Tombusviridae***. The PMV genome map as shown in Figure 1. The amino acids (aa) 306–405 (speckled region) represent a conserved domain (CD), common to the analogous *Tombusviridae *proteins, as determined by BLAST-PSI and manual alignment. The percent identities for this region are indicated on the left and the virus abbreviations are defined in the Methods. The amino acids are given in single-letter code. Alanine-scanning mutagenesis was targeted to ten amino acids on the PMV genome selected from the consensus sequence.

Further sequence comparisons revealed a protein domain spanning approximately 100 amino acids located upstream from the PMV p48 read-through stop codon (Fig. 4). In PMV, this conserved domain (CD) is located between residues 306–405 (Fig. 4). This domain was common to four genera (*Panicovirus*, *Machlovirus*, *Carmovirus, Necrovirus*) and amino acid identity values in this region ranged from 29–43% (Fig. 4) [2,17,19]. Some of these amino acids also were present in the corresponding proteins encoded by members of the *Avenavirus *(OCSV), *Tombusvirus *(TBSV), and the *Dianthovirus *(RCNMV) genera.

### Specific amino acid substitutions in the conserved domain (CD) affect virus accumulation in protoplasts

First, we investigated if the CD was important for PMV replication in protoplasts. Although SPMV replicates *in trans *when co-inoculated with PMV, wild type virus replication and infection of plants requires p48 and p112 expression *in cis *(Figs. 1 and 2). From this we realized that it would be imperative to use the full-length infectious cDNA of PMV for biological assays of the CD-amino acid mutants on plants. The CD alanine scanning mutagenesis did not affect translation of either p48 or p112 based upon *in vitro *translation of PMV gRNA in wheat germ extracts (data not shown).

**Figure 5:**
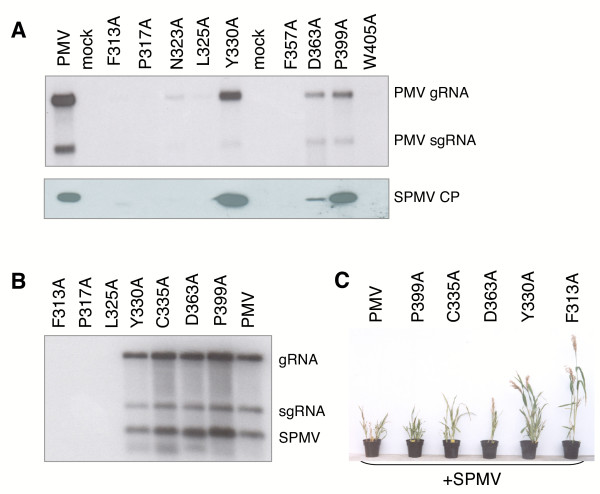
**Replicase motif mutations to the conserved domain (CD) affect PMV replication in protoplasts and plants**. **(A) **Foxtail millet protoplasts were transfected with transcripts of alanine replicase mutants (Fig. 4) and harvested 40 hours postinoculation. The RNA was extracted and separated on 1% agarose gels, blotted, and probed for PMV accumulation with a ^32^P-labelled cDNA that detects genomic (g) and subgenomic (sg) RNA. Proteins were separated via SDS-PAGE and probed with rabbit polyclonal antiserum against the SPMV coat protein (CP) (bottom). **(B) **Proso millet plants were mechanically co-inoculated with SPMV and either PMV or the replicase mutants. The blots were probed for PMV, as described for panel A, and then hybridized with a ^32^P-labelled SPMV cDNA. **(C) **Proso millet plants at 1-month postinoculation with PMV+SPMV or the replicase motif mutants (P399A, C335A, D363A, Y330A, and F313A) plus SPMV.

Each mutant was tested in foxtail millet (*Setaria italica *cv. German R) protoplasts to examine the effects of amino acid substitutions on PMV replication. Transcripts of the CD-mutants were co-inoculated with SPMV to determine if an amino acid substitution moderated sequence-independent *trans*-replicating molecules. The results showed that disruption of several conserved amino acids had a significant effect on PMV RNA (Fig. 5A) and capsid protein (data not shown) accumulation in protoplasts (Fig. 5A). Four mutations (F313A, L325A, F357A, and W405A) were replication incompetent in protoplasts, based on a lack of detectable PMV RNA. Conserved domain amino acid mutants P317A and N323 replicated poorly and inconsistently. PMV mutants C335A, D363A, and P399A and Y330A were replication competent and supported SPMV replication in protoplasts.

### Replication-competent CD mutations variably affect PMV and SPMV accumulation in millet plants

The same ten alanine-scanning mutants were tested on foxtail millet and proso millet (*P. miliaceum *cv. Sun Up) plants. Proso millet is permissive for higher levels of PMV accumulation and exhibits more severe symptoms than similar infections in foxtail millet plants [8]. PMV mutants were tested alone or as mixtures with SPMV. Co-inoculations of PMV+SPMV causes a severe mosaic and stunting in systemically infected plants that is primarily determined by the SPMV CP [7,21,22].

As observed for protoplasts, CD amino acid mutants F313A, F357A, and W405A were lethal for replication of PMV +SPMV in plants, as determined by the lack of detectable accumulation of helper virus and satellite virus (Fig. 5). Replication competent mutants Y330A, C335A, D363A, and P399A (Fig. 5A) consistently developed systemic infections in plants (Figs. 5B and 5C), but the relative accumulation was variable. All replicating mutants also supported systemic infections of SPMV (Fig. 5), although the infections were delayed, compared to wild type PMV+SPMV infections.

### Plant-dependent effects of CD mutants on systemic invasion and symptom development

**Figure 6:**
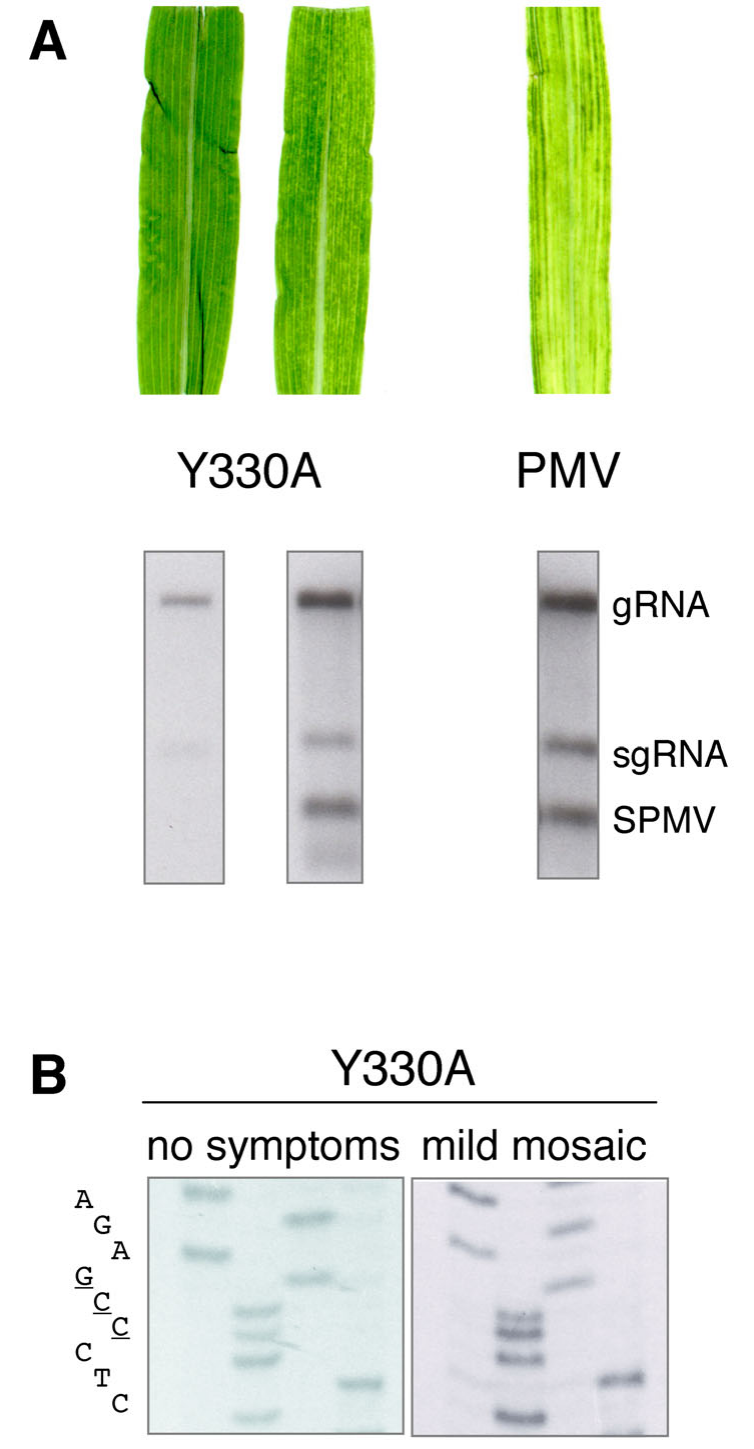
**Symptom responses and replication observed during mixed infections of Y330A plus SPMV on proso millet**. **(A) **Y330A+SPMV-infected plants with no obvious symptoms (left leaf) or mild mosaic symptoms (right leaf). A leaf from a PMV+SPMV infected plant is also shown. RNA isolated from plants was used for RNA blots to detect PMV genomic (g) and subgenomic (sg) RNAs and SPMV RNA. **(B) **RT-PCR clones were sequenced to check the stability of the Y330A mutation. The tyrosine (TAC) to alanine (GCC) mutation on the PMV cDNA was stable in Y330A-infected plants with mild or severe symptoms, as shown in panel A.

We also found that some of the plants co-inoculated with SPMV and viable CD mutants showed milder symptoms and effects on plants than typically associated with PMV+SPMV infections (Fig. 5C). The mild symptoms (and delayed systemic spread) were likely due to a reduced accumulation of PMV; this in turn reduced the accumulation of SPMV and its CP, which is the main symptom determinant [21,22]. The effect was a less striking phenotype as illustrated for Y330A in Fig. 6A. In contrast, infected plants with more severe symptoms had higher levels of both virus and satellite virus, at levels comparable to wild type infections (Fig. 6A).

We considered that the severe symptoms and increased virus accumulation observed in some plants might be due to a CD-mutation reverting to wild type. To exclude this possibility, we cloned and sequenced viral RT-PCR products from plants inoculated with the replicating mutants that had variable symptoms (Y330A, C335A, D363A, and P399A). All four of the mutations were stably maintained. For example the alanine residue is maintained for Y330A cDNA re-isolated from plants that were either symptomless or displayed mild mosaic symptoms (Figs. 5 and 6). Identical results were obtained for the other three replicating mutants (C335A, D363A, and P399A; data not shown).

Symptomatic tissue from each mutant (Fig. 5C) was rub-inoculated to healthy millet to evaluate if passage would affect symptom development or virus accumulation. Symptom development was delayed by one or more days in plants inoculated with each mutant compared to wild type infections. At one month post-inoculation, plants re-inoculated with wild type PMV+SPMV were severely stunted. Plants inoculated with the mutants displayed symptoms that reflected the original phenotypes, as exhibited in Fig. 5C.

The collective results obtained for replication-competent CD mutants, exemplified by Y330A (Figs. 4, 5, and 6), shows that the CD mutations are stable and maintained by passage in plants. Interestingly, in some plants the mutants induced severe symptoms and accumulated to levels similar to that observed for wild type (Figs. 5B and 5C) whereas in other plants symptoms were mild and had lower titers (e. g. Y330A, Figs. 5 and 6). Therefore, it appears that some undefined host or environmental variable(s) leads to differences in systemic infection and symptom development between plants.

### CD mutations do not affect RNA *cis*-elements

**Figure 7:**
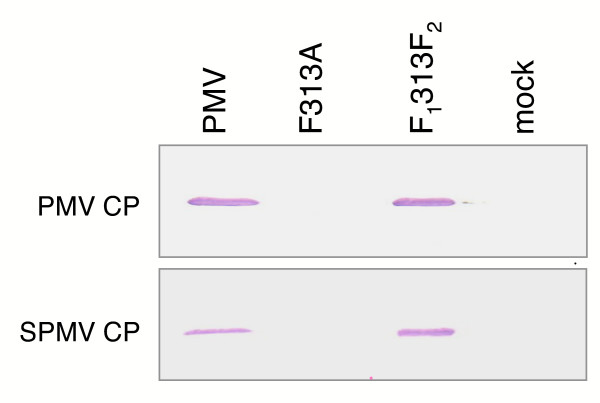
**Phenylalanine residue 313 on the conserved domain is required for PMV and SPMV replication in millet plants**. Proso millet plants were co-inoculated with transcripts of PMV or its phenylalanine mutants (F313A and F_1_313F_2_) plus SPMV and screened for the respective coat proteins by immunoblot assay. F313A contained a change in the amino acid while F_1_313F_2 _contained a change in the encoding RNA at the same position but maintained the wild type phenylalanine residue.

Many RNA viruses contain RNA *cis*-elements that can affect replication. To test if amino acid changes (F313A, F357A, and W405) may have inactivated an RNA element, we mutated F313A (Table 1, Fig. 4) from the PMV cDNA codon (TTC) to TTT creating a new mutant F_1_313F_2_. While F313A is lethal (Figs. 5 and 7), this new mutation (F_1_313F_2_) slightly changed the RNA sequence while maintaining the phenylalanine codon. We co-inoculated F313A or F_1_313F_2 _transcripts with SPMV to proso millet and compared them with wild type PMV infections. As expected, F313A inoculated plants were asymptomatic and lacked viral RNA (Fig. 5) and CP (Fig. 7). In contrast, the F_1_313F_2 _mutant (Fig. 7) accumulated to wild type levels with a typical systemic mosaic on infected plants. This result helps support our hypothesis that the conserved replicase amino acids, and not the encoding RNA, are necessary for replication of PMV and SPMV in host plants.

**Table 1:** Mutagenesis primers used to examine the role of the PMV replicase motif in virus accumulation.

Mutant	Primer	Sequence (5'-3')^a^
F313A	REP/F1-A	CCCCAGCGGCTTCGTTCTTTGC
F1313F2	F1-313-F2	GGAACCCCAGCAAACTCGTTCTTTGC
P317A	P317A-989R	CTGTGGGTTTTGCAACCCCAGCG
N323A	N323A-1007R	CAGCCAACTGGGCAGCCTCTGTG
L325A	L325A-1014R	CTCCAGACAGCCGCCTGGTTAGC
Y330A	REP/Y-A	CACACCCTGTAGAGGGCTCTCCAG
C335A	1044R-C/A	CCTTTCTTATCAGCCACCCTGTAGAG
F357A	REP/F-A	GGAATACAGCTGGCAAGGC
D363A	REP/D-A	TGATCCTGGGCGTATGCGC
P399A	MUTPMV-1236R	GCCCCAACTAATGCATTGGTCACTAG
W405A	REP/W-A	CCAAGCAGTCGCATTGGCCCC

a. The altered nucleotides on the PMV cDNA are underlined.

## Discussion

Within the *Tombusviridae*, PMV is one of the few well-characterized carmo-like viruses that infect monocots [2,18]. Because PMV also supports a satellite virus, satellite RNAs [1,6,7], and a satellite virus-derived DI [8,9], it is an excellent model to study *cis*- (for PMV) and *trans*- (for subviral agents) replication elements. In this study we examined the role of p48 and p112 and the defined CD in replication and pathogenicity of PMV and SPMV.

Biochemical fractionation experiments showed that both p48 and p112 are associated with membrane-enriched fractions, and these fractions have *in vitro *RdRp activity. This is similar to what has been observed for other members of *Tombusviridae *in dicot plants. Thus, our findings suggest that the replication complex of monocots bears a strong resemblance to this process on dicotyledonous plants. In combination with earlier electron microscopy studies that showed the presence of vesicle-like structures in PMV-infected millet cells [23], we suggest that the replication of PMV occurs in membranous p48- and p112 enriched vesicle-like structures. These complexes may functionally resemble those recently defined for *Brome mosaic virus *[24,25].

PMV and MCMV differ from other carmo-like viruses of the *Tombusviridae*, including TCV and *Tobacco necrosis virus *(TNV) that are defined by smaller replicase ORFs of 28 kDa and 33 kDa, respectively. The PMV p48 ORF has a 19 kDa N-terminal extension that does not have sequence homology with other viral proteins. The C-terminal portion the PMV p48 ORF contains sequence homology to other 5'-proximal *Tombusviridae *replicase ORFs. *In vitro *translation of PMV genomic RNA in wheat germ extracts results in the production of p48, p112, and a ~30 kDa protein (p48C), indicating the possibility of internal initiation of translation. The sequence p48C shares similarity by size to carmo-like RdRp proteins, suggesting it might have a functional role in PMV replication. One possibility is that the p48C protein is generated by internal initiation from an in-frame AUG start codon (nt 545) downstream of the authentic p48/p112 start codon at nt 29, resulting in generation of a 29 kDa protein (and a read-through product). However introduction of a stop codon immediately downstream of the p48 AUG, abolished replication in protoplasts [19]. From this, p48C and its read-through product are not independently active replicase-associated products.

Alternatively, the N-terminal portion of p48 (and/or p112) may be involved in membrane targeting, and this portion would be imbedded in the host membranes. In support of this, we have identified a putative type 2 peroxisome targeting sequence (PTS2) in the N-terminal region of the PMV p48 protein. We hypothesize that the p48C portion of the protein (and perhaps its putative 93-kDa read-through product) represent truncated RdRp proteins produced through cleavage *in planta*. In support of this assumption, we detect p48C in the cytosol.

Results of replication assays in protoplasts with transcripts of pKB238 and pQP94 validate the conclusion that the CP and movement-associated genes of PMV are dispensable for replication. However, the somewhat reduced levels of QP94 compared to KB238 RNA accumulation suggest that the p8 protein has an auxiliary role. Similarly, a slight negative effect on RNA accumulation was also observed upon the inactivation of the movement protein gene of TBSV [26]. In addition, our results show that PMV-encoded p48 and p112 are sufficient for *trans*-replication of SPMV. This observation is comparable the ability of the TNV replicase genes expressed in transgenic plants to support replication of its satellite virus, STNV [27].

Experiments show that PMV replication requires both the 48-kDa (pRT-Stop) and 112-kDa (pAmb-Tyr) proteins. These results are similar to what has been reported for dicot-infecting viruses in the *Tombusviridae *[10,16,28]. We also determined that in mixed co-transfections of pAmb-Tyr and pRT-Stop transcripts complemented one another to restore PMV replication in protoplasts. However, this sort of co-inoculation was not viable in plants, suggesting that regulation of the sgRNA had been perturbed. This, in turn, would affect the expression of movement-associated proteins. The implication is that replication of PMV gRNA and SPMV RNA can occur *in trans*, but that sgRNA transcription is a *cis*-regulated event.

### Involvement of the PMV CD in replication and pathogenesis

As we had first identified in a preliminary report [17] the replicase proteins of members in the *Tombusviridae *have a conserved domain (CD) (Fig. 4). The effects of CD amino acid substitutions could be divided into three distinct replication phenotypes: lethal, severely impaired, and competent. First, there are the effects of changing hydrophobic amino acids to alanine. In such cases (P313A, P317A, N323A, L325A) the CD-domain mutants were incompetent for replication in whole plants. In protoplasts, these mutants replicated poorly or RNA accumulation was not detected. The affected amino acids of these mutants may contribute to localization to anchor the RdRp complex to host membrane(s).

When mutants Y330A or D363A, that accumulate to wild-type levels in plants (Fig. 5B), were co-inoculated with SPMV onto plants, mosaic and mottling on emerging leaves appeared approximately 2–5 days after wild type PMV+SPMV infections on both proso and foxtail millet. In addition, plants infected with these mutants in the presence of SPMV were generally not as stunted as those infected with wild type PMV+SPMV (Fig. 5C). The difference between the delayed mutants and wild type PMV was more obvious in foxtail millet, which is more restrictive to SPMV movement [8]. This supports our previous observations that the accumulation of SPMV CP is the primary determinant for severe symptoms in foxtail millet plants [22].

In general, millet plants infected with PMV+SPMV develop severe symptoms, including mosaic and stunting (Fig. 5C) [7]. In contrast, symptoms in plants infected with CD-mutants plus SPMV ranged from mild to severe and the severity was directly correlated with the amount of the virus and satellite virus in each plant. Yet in plants with mild symptoms or severe symptoms, following infection with the same CD-mutant virus, the mutations were stable and no reversion had occurred. Thus, CD mutations had unpredictable plant-dependent effects on the systemic invasion and symptom presentation on individual plants.

In conclusion, we have demonstrated that p48 and p112 of the monocot-infecting PMV are required and sufficient for replication and that specific amino acids in the CD region play an essential role in this process. The hydrophobic amino acids within this domain appear to be particularly important as replication determinants, possibly by directing the replicase complex to cellular membranes. Other residues on the CD contribute to systemic invasion in a complex manner that might be related to movement and overcoming defense mechanisms.

## Methods

### Sequence analysis/alignment of p48

The PMV p48 ORF was used to search GenBank using BLASTP and BLAST-PSI [29,30] to generate a preliminary alignment of homologous proteins. The *Tombusviridae *consensus sequence was identified by comparing the 5'-proximal replicase proteins from representative members of the *Tombusviridae*: *Panicum mosaic virus *(PMV; Accession# U55002), *Maize chlorotic mottle virus *(MCMV; X14736), *Galinsoga mosaic virus *(GaMV; Y13463), *Melon necrotic spot virus *(MNSV; M29671), *Turnip crinkle virus *(TCV; M22445), *Cardamine chlorotic fleck virus *(CCFV; L16015), *Carnation mottle virus *(CarMV; X02986), *Tobacco necrosis virus *(TNV-A; M33002), *Oat chlorotic stunt virus *(OCSV; X83964), *Red clover necrotic mosaic virus *(RCNMV; J04357), and *Tomato bushy stunt virus *(TBSV; M31019).

### Alanine scanning site-directed mutagenesis of the PMV cDNA

Single-stranded DNA was generated from pPMV85, a full-length infectious cDNA construct [2] and used as a template for site-directed mutagenesis [31]. Ten mutagenic oligonucleotide primers (Table 1) were designed to change codons for individual conserved amino acids into those specifying alanine. Mutations were confirmed by sequence analysis as described previously [2].

### Characterization of the p48 and p112 as replicase genes

The full-length PMV cDNA was modified to abolish the sgRNA-encoded genes to determine if p48 and p112 were sufficient for replication. The plasmid pKB238 had an *ApaI *fragment deletion from nucleotides 3129 to 3400. This abolished the expression of p6.6, p15, and the CP genes. In addition, pMAX6, a previously described construct that abolished the expression of the p8 gene [18], was digested with *Apa*I and religated. This construct, pQP94, expressed p48 and p112, but not the genes encoded on the sgRNA. A second set of constructs, pAmb-Tyr and pRT-Stop were designed to express p48 or p112, respectively (Fig. 1).

### Fractionation of a membrane-bound PMV replicase complex

The PMV replicase purification procedure was modeled after that used for purification of *Cucumber mosaic virus *[32]. Proso millet (*Panicum miliaceum *cv. Sun Up) plants were mechanically inoculated with approximately 15 μg of uncapped PMV transcripts in inoculation buffer (0.05 M K_2_HPO_4_, 0.05 M glycine, 1% bentonite, 1% Celite, pH 9.0) [2]. PMV-infected leaves were harvested at 6–10 days post inoculation (dpi) [2].

The upper, non-inoculated leaves were collected, chopped, and blended in four volumes of buffer A [50 mM Tris-HCl, pH 8.0, 15 mM MgCl_2_, 10 mM KCl, 20% glycerol v/v, 10 mM dithiothreitol (DTT) and 1 mM phenylmethylsulfonyl fluoride (PMSF)]. This total extract was filtered through 4 layers of cheesecloth and centrifuged at 1000 × *g *for 10 min at 4°C. The pellet (P1, large organelle fraction) was resuspended in buffer B (50 mM Tris-HCl, pH 8.0, 15 mM MgCl_2_, 1 mM DTT, 1 mM PMSF, and 5% glycerol v/v), and centrifuged at 44,000 × *g *for 45 min. The pellet (P44, membrane fraction) was resuspended in Buffer B and stored at -80°C, along with the supernatants for RdRp assays or further purification. A portion of the supernatant (S100) was further concentrated by precipitation with four volumes of cold ice-acetone, 85% ammonium sulfate-saturated solution, or by microfiltration (300,000 nominal molecular weight limit) (Millipore, Bedford, MA) for protein assays.

### RdRp assay

Ten μl of each fraction were added to a reaction mix containing 2× RdRp buffer (100 mM Tris-HCl, pH 8.0, 20 mM MgCl_2_, 8% glycerol v/v, 2 mM ATP, 2 mM CTP, 2 mM GTP, 50 μM UTP, 20 mM DTT), 1 unit of RNase inhibitor (SUPERase-In; Ambion, Austin, TX), and 1–3 μl [^32^P]-UTP (10 mCi/ml) [32]. The reaction was incubated for 1 hr at 30°C and the newly synthesized RNA was extracted with an equal volume of phenol:chloroform:isoamyl alcohol (25:24:1) followed by ethanol precipitation. The RNA was resuspended in 25 μl TE (10 mM Tris-HCl, 1 mM EDTA, pH 8.0) containing SUPERase-In and 10 μl were analyzed on 1% agarose gels and vacuum-dried for autoradiography to detect [^32^P]-UTP incorporation into newly synthesized RNA products.

### Detection of PMV and SPMV RNA and proteins in millet plants and protoplasts

Transcripts of each PMV-derived mutant were inoculated to foxtail millet (*Setaria italica *cv. German R) and proso millet plants grown in the greenhouse (28°C to 30°C) or in the growth chamber (28°C, 14 h of light; 24°C, 10 h dark). Plasmid DNA containing the SPMV genome was linearized with *Bgl*II prior to *in vitro *transcription [7]. Inoculated millet plants were maintained in the greenhouse or growth chamber and monitored visually for symptom development and screened for virus replication and protein accumulation at several time points.

Mutants were also tested in foxtail millet protoplasts. Protoplasts were isolated from 10–14 day old plants, as described previously [33], except that protoplasts were centrifuged at 210 × *g *(instead of 70 × *g*). Approximately 10^6 ^protoplasts were transfected with ca. 6 μg uncapped transcript and incubated in a growth cabinet (28°C, 14 h light; 24°C, 10 h dark) for 40 to 48 h prior to protein or RNA extraction.

Total RNA and protein were extracted from 100 mg of inoculated or mock-inoculated leaves bulked from four plants 8–14 days post-inoculation or from protoplasts using 1× STE buffer (10 mM Tris-HCl, 10 mM NaCl, 1 mM EDTA, pH 8.0) containing 1% SDS. One-half of the extract was combined with an equal volume of sample buffer (1.3 M Tris, pH 6.8, 5% SDS, 5% β-mercaptoethanol, 5% bromophenol blue, and 50% glycerol), boiled, and separated by electrophoresis through a sodium dodecyl sulfate-12.5% polyacrylamide gel (SDS-PAG) and transferred to nitrocellulose membrane. Membranes were probed with rabbit polyclonal anti-PMV or anti-SPMV coat protein antisera as described previously [7]. The secondary goat-anti rabbit IgG conjugated with alkaline phosphatase (Sigma-Aldrich, St. Louis, MO) or horseradish peroxidase (Amersham Pharmacia Biotech, Piscataway, NJ) was used at a 1:5,000 dilution and assayed by enzymatic reactions. The remaining half of the extract was prepared for RNA blots, as described previously [7].

## Competing interests

The author(s) declare that they have no competing interests.

## Authors' contributions

The authors each contributed equally to the experimental design, analysis, and writing of this manuscript. All authors have approved the final version of this manuscript.
